# Unconscious and Conscious Gaze-Triggered Attentional Orienting: Distinguishing Innate and Acquired Components of Social Attention in Children and Adults with Autistic Traits and Autism Spectrum Disorders

**DOI:** 10.34133/research.0417

**Published:** 2024-07-10

**Authors:** Fang Yang, Junbin Tian, Peijun Yuan, Chunyan Liu, Xinyuan Zhang, Li Yang, Yi Jiang

**Affiliations:** ^1^ State Key Laboratory of Brain and Cognitive Science, Institute of Psychology, Chinese Academy of Sciences, Beijing, P.R. China.; ^2^ Department of Psychology and College of Life Science, University of Chinese Academy of Sciences, Beijing, P.R. China.; ^3^ Peking University Sixth Hospital, Peking University Institute of Mental Health, National Clinical Research Center for Mental Disorders (Peking University Sixth Hospital), NHC Key Laboratory of Mental Health (Peking University), Beijing, P.R. China.; ^4^ School of Education and Psychology, University of Jinan, Jinan, P.R. China.; ^5^ School of New Media, Financial & Economic News, Guangdong University of Finance, Guangzhou, P.R. China.

## Abstract

Typically developing (TD) individuals can readily orient attention according to others’ eye-gaze direction, an ability known as social attention, which involves both innate and acquired components. To distinguish between these two components, we used a critical flicker fusion technique to render gaze cues invisible to participants, thereby largely reducing influences from consciously acquired strategies. Results revealed that both visible and invisible gaze cues could trigger attentional orienting in TD adults (aged 20 to 30 years) and children (aged 6 to 12 years). Intriguingly, only the ability to involuntarily respond to invisible gaze cues was negatively correlated with autistic traits among all TD participants. This ability was substantially impaired in adults with autism spectrum disorder (ASD) and in children with high autistic traits. No such association or reduction was observed with visible gaze cues. These findings provide compelling evidence for the functional demarcation of conscious and unconscious gaze-triggered attentional orienting that emerges early in life and develops into adulthood, shedding new light on the differentiation of the innate and acquired aspects of social attention. Moreover, they contribute to a comprehensive understanding of social endophenotypes of ASD.

## Introduction

In social interaction, eye gaze provides a wealth of information about an individual’s focus of attention, mental state, implicit intention, etc. [1,2]. Extensive evidence suggests that observing a directional eye gaze can trigger a shift of attention toward the gazed-at location [3–6]. This ability, also known as social attention [7] or joint attention [8], emerges early in life and is fundamental to the development of more complex social abilities [9], including language [4,5] and theory of mind [10,11].

Despite the theoretical and practical importance of gaze-triggered social attention, its nature remains elusive. It has been proposed that there might exist a quick eye direction detector [3] or an innate social module [12] that detects eye-like stimuli in the environment and orients attention accordingly. Evidence supporting this view has found that gaze-triggered social attention is reflexive [13,14], independent of consciousness [15,16], and heritable [17]. Moreover, various nonhuman animals, such as apes [18], monkeys [19], wolves (and dogs) [20,21], and birds [22,23], exhibit a similar social attention ability. The accumulating evidence suggests that this ability may be “hard-wired” in the vertebrate brain, similar to the predisposition to attend to face and biological motion [24].

On the other hand, social attention can also be shaped by learned social strategies developed through repeated exposure to gaze direction and its association with “interesting sights” [25]. In line with this, some studies show that responding to eye gaze in infancy is influenced by social experience [26,27]. Furthermore, social attention in adulthood resembles endogenous attention as it persists over a relatively long interval [28] and can be modulated by multiple social factors [29]. It has therefore been proposed that the emergence and development of the gaze-triggered social attention might arise from the mutual contributions of the innate social module [3,12] and the reinforcement learning through social experience [30,31] or perceptual association [32]. Indeed, recent studies have demonstrated that social attention triggered by gaze cues not only is supported by innate and genetically inherited mechanisms tuned to social processing [17,33] but also heavily relies on learned and general attentional mechanisms shared by nonsocial processing (e.g., arrow cues). However, it is difficult to dissociate the respective contributions of the innate and acquired components to gaze-triggered attentional orienting using a gaze-cueing paradigm.

This difficulty also poses an obstacle to understanding the impairments of social attention skills in autism spectrum disorder (ASD), a heritable disorder characterized by qualitative deficits in social interaction [34]. Despite evident clinical findings of impaired social attention skills in people diagnosed with ASD [8,35], studies adopting the typical gaze-cueing paradigm often observed undistinguished attentional orienting effects or gaze-following behaviors between autistic individuals and typically developing (TD) individuals [36–38]. It has been postulated that people with ASD may achieve comparable performance in traditional social attention tasks through nonsocial strategies [39], such as by responding to the physical features of the stimuli (e.g., eye movement) or by treating gaze as a learned nonsocial symbol (e.g., an arrow). These observations lead to the conjecture that the gaze-cueing paradigm might not be sensitive enough to precisely pinpoint the locus of the impaired social attention ability in ASD [40,41].

To address these issues, the present study aims to isolate the innate and involuntary component of gaze-triggered social attention through an unconscious paradigm. Accumulating evidence suggests that face and gaze stimuli rendered invisible through unconscious paradigms either are insufficient to evoke cortical responses or evoke weaker responses than visible stimuli, while they can evoke equal or even stronger responses in subcortical structures [9,42,43]. These subcortical structures involve evolutionarily conserved nuclei, such as the amygdala, superior colliculus, and pulvinar, which also contribute to innate attentional bias about biologically important social information [44,45]. In contrast, cortical structures supporting social attention generally contribute more to the experience-based processing of face information [46,47] and top-down regulation of attention [48–50]. Therefore, unconscious paradigms were proposed as an effective way to minimize the information transmission through cortical pathways while retaining the subcortical contribution [51,52].

We adopted a technique referred to as “critical flicker fusion” (CFF) to render the gaze direction “invisible” (or unperceivable to consciousness) and minimize the influence of consciously acquired strategies. In this technique, two oppositely colored stimuli were alternately presented at a temporal frequency above the flicker fusion threshold (~25 Hz) [53–55] so that they would fuse into one uniform color. Despite the perceptual sensation of color fusion, color opponent cells at early stages of the visual pathway from retinal to visual cortices can still respond to flicker exceeding the perceptual fusion threshold [56–58]. Functional magnetic resonance imaging studies on humans and macaques found that CFF stimuli elicit stronger responses than stable colors in the visual cortex [59,60] but not in frontoparietal cortical areas [61], due to the limited temporal resolution of higher cortical areas [62]. This technique was widely used in previous research on unconscious visual processing [59,63–65].

Moreover, the CFF technique provides several advantages over the traditional masking method in manipulating participants’ conscious awareness of the stimuli. First, it allows the visible and invisible stimuli to be presented to both eyes for exactly the same duration. More importantly, it does not involve any additional mask or noise stimulus that might substantially distract the attention of individuals with ASD or high autistic traits [66,67]. Hence, it offers a suitable means to explore the attentional orienting in both TD and ASD participants.

**Fig. 1.**
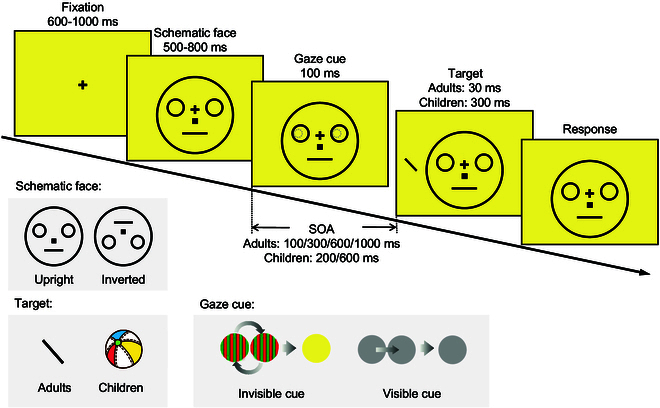
Illustration of stimuli and procedure for all experiments. During the experiments, participants were presented with an upright or inverted schematic face on the screen for a duration that varied across trials. They were instructed to maintain their gaze on the central cross positioned between the eyes of the face. Subsequently, the pupils within the eyes appeared, indicating a gaze direction either toward the left or right. In the invisible condition (experiments 1, 2, and 3), the pupils were represented by two anti-phased red-and-green sinusoidal grating discs that alternated at frequencies of 30 Hz. In the visible condition (experiments 4, 5, and 6), the pupils were depicted as two constant gray patches. Following a variable interval, a target stimulus (a tilted line or colored ball) randomly appeared on either the left or right side of the face. Participants were given instructions to respond as quickly and accurately as possible by pressing corresponding keys to indicate the tilted direction (for adults) or the location (for children) of the target. Please note that the color and size of the stimuli depicted in the figure are for demonstration purposes only and may differ from the actual stimuli used in the experiments.

In the current study, we combined the CFF technique with a gaze-cueing paradigm, where subjects were required to detect a target that appeared randomly on either side of a schematic face (Fig. 1). The pupils of the schematic face, which presented in positions shifted to the left or right to constitute gaze directions, were rendered invisible through the CFF method. The gaze-cueing effect (GCE), with faster responses to targets that were congruent with the gaze direction compared to incongruent targets, was adopted as a behavioral index of whether subjects followed the gaze cues.

First, we investigated whether such invisible gaze cues could trigger substantial attentional orienting in TD adults (experiment 1a) and whether a similar effect could be observed with invisible nonsocial arrow cues (experiment 1b). Then, we explored whether the unconscious gaze-triggered social attention ability was impaired in adults diagnosed with ASD (experiment 2). Furthermore, we generalized the effects in children aged between 6 and 12 years (experiment 3) and further verified the association between the unconscious gaze-triggered attentional orienting and autistic traits. Last, we examined and compared the social attention effect induced by visible gaze cues with that induced by invisible gaze cues in TD and ASD adults and children (experiments 4, 5, and 6). Based on these empirical findings, we can extend previous findings and more comprehensively delineate the nature of the unconscious and conscious gaze-triggered social attention as well as their respective relationship with autistic traits and ASD.

## Results

We first verified that the participants were completely unaware of the invisible cues. During the unconscious sessions, all the included participants reported that they saw nothing within the eye areas in the context of the schematic face during the main task. Moreover, the participant’s performance in localizing the invisible cues in the forced-choice task did not significantly deviate from chance level at both individual (binomial test, *P*s > 0.05) and group levels [experiment 1a: mean = 49.75%, SD = 4.82%, *t*(34) = −0.31, *P* = 0.761, Cohen’s *d* = −0.05; experiment 1b: mean = 50.67%, SD = 5.11%, *t*(29) = 0.71, *P* = 0.481, Cohen’s *d* = 0.13; experiment 2: mean = 51.92%, SD = 4.91%, *t*(12) = 1.41, *P* = 0.183, Cohen’s *d* = 0.39; experiment 3: mean = 51.25%, SD = 6.28%, *t*(33) = 1.16, *P* = 0.254, Cohen’s *d* = 0.20]. The results of both subjective and objective awareness check tasks indicated that the participants could not detect the existence of the invisible cues and were therefore unaware of the cue direction.

### Unconscious gaze-triggered social attention in TD adults

In experiment 1a, we probed the attentional effect induced by invisible gaze cues and manipulated the orientation of the contextual schematic face (upright versus inverted) to examine whether the observed cueing effect is indeed induced by the perceived gaze direction rather than the low-level visual properties, such as the physical position of the cues. Based on previous findings, the inverted face preserved the low-level visual properties of the upright face but with its integrated social processing disrupted [68]. To determine the time course of the unconscious social attention, we set 4 different time intervals (100, 300, 600, and 1,000 ms) used in previous studies [14] as stimulus onset asynchrony (SOA) conditions. The GCE induced by visible gaze cues emerged as early as 200 ms and could extend up to 800 ms, a time course considered one of the special characteristics of social attention [69]. A three-way analysis of variance (ANOVA) (face orientation × cue congruency × SOA) on mean reaction time (RT) revealed no significant main effect of face orientation [*F*(1,34) = 1.45, *P* = 0.237, *η^2^_p_* = 0.04] or cue congruency [*F*(1,34) = 0.37, *P* = 0.550, *η^2^_p_* = 0.01], but a significant two-way interaction of face orientation × cue congruency [*F*(1,34) = 4.63, *P* = 0.039*, *η^2^_p_* = 0.12] and a three-way interaction [*F*(3,102) = 2.84, *P* = 0.042*, *η^2^_p_* = 0.08]. No other interactions reached significance (all *F*s < 2.0, *P*s > 0.100).

**Fig. 2.**
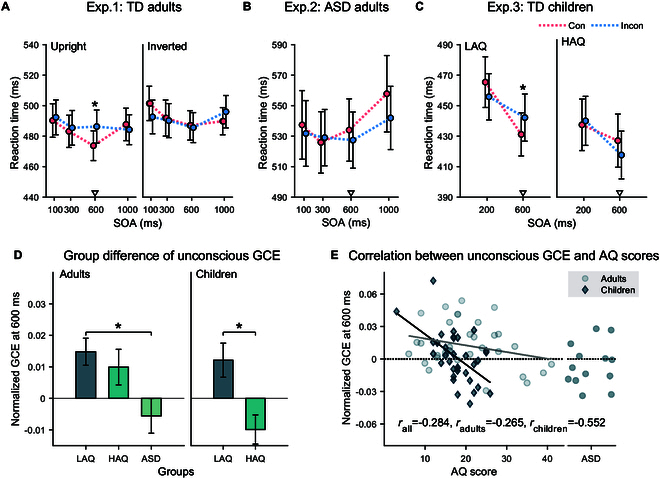
Unconscious GCE and its relationship with autistic traits and ASD. (A) When the schematic face was upright (left plane), the invisible gaze cues effectively induced a cueing effect at the 600-ms SOA level (~12 ms). However, this effect disappeared when the schematic face was presented upside-down (right plane). (B) The unconscious GCE did not emerge in autistic adults. (C) The children with low autistic traits (LAQ) exhibited a significant cueing effect at the SOA of 600 ms, while the children with high autistic traits (HAQ) did not show this effect. (D) The bars show the unconscious GCEs of different groups at the SOA of 600 ms in both adults and children. (E) The scatterplots demonstrate the correlations of the unconscious GCE at the SOA of 600 ms with AQ scores in TD children (represented by dark diamond) and TD adults (represented by light dots). The individual data for autistic adults are labeled on the right side of the scatter for reference. The children group showed a significantly moderate negative association, while the adults group exhibited a negative correlation trend. The triangle markers in the line graph indicate the levels of SOA used for between-group analysis of the GCEs. Error bars represent SEM. *P < 0.05, corrected for multiple comparisons.

To clarify how the face orientation affected the unconscious gaze-induced attentional effects across different SOAs, we further conducted the two-way ANOVAs for the upright and inverted face conditions, respectively. As shown in Fig. 2A, we found a significant interaction of cue congruency × SOA in the upright face condition [*F*(3,102) = 3.03, *P* = 0.033*, *η^2^_p_* = 0.08]. Simple effect analysis revealed that the cueing effect was present at 600 ms [474 ms versus 486 ms; *F*(1,34) = 11.89, *P*_Sidak_ = 0.002*, *η^2^_p_* = 0.26], suggesting that this attentional orienting emerged at a relatively longer SOA. In contrast, there was no significant main effect or interaction in the inverted face condition (all *F*s < 2.3, *P*s > 0.100). Consistent with previous findings [15], these results demonstrated that invisible gaze cues could trigger unconscious attentional orienting. The modulation effect of the face orientation further suggests that this involuntary attentional orienting essentially relies on the biosocial context of the gaze cues rather than the mere positional shifts of the invisible eyes that were present in both the upright and inverted face conditions.

In experiment 1b, we investigated the potential attentional effects of arrow cues rendered invisible through the CFF technique within a similar schematic face (see Fig. S1). Previous research has demonstrated a similarity between the attentional effects induced by gaze cues and arrow cues [70–72]. Particularly, when presented as schematic faces, gaze cues produce behavioral effects that are nearly indistinguishable from those generated by arrow cues in TD individuals [29]. This similarity has led many studies to utilize arrow cues as control stimuli to examine the biological specificity of gaze-triggered attentional orienting (e.g., [73–76]). However, different from the invisible gaze cues, the invisible arrow cues did not reveal any significant attentional effects (see Supplementary Results). These findings suggest that the attentional effect induced by the unconscious gaze cue may be specific to the biosocial context and contingent upon the distinctive morphology of the eyes [77].

In addition, all the experiments showed a significant main effect of SOA (*F*s > 4.00, *P*s < 0.050) on RT, reflecting a potential preparation effect [78] that was not related to the focus of the present study.

### No evidence of unconscious gaze-triggered social attention in adults with ASD

Experiment 1 demonstrated that the invisible schematic gaze cues could induce a prominent social attention effect in TD adults at the SOA of 600 ms. To determine if the ability to involuntarily respond to invisible gaze cues is intact in ASD, we recruited autistic adults and analyzed the group differences in the unconscious GCE. Since the GCE was only found under the upright condition in experiment 1, we only conducted the upright condition in the ASD group to improve the efficiency. A two-way ANOVA (cue congruency × SOA) found that neither the main effect of cue congruency nor its interaction with SOA was significant (*F*s < 2.3, *P*s > 0.100), suggesting that invisible gaze cues failed to trigger an attentional shift in the ASD group (Fig. 2B).

Considering that the unconscious GCE in TD adults was only evident at the SOA of 600 ms, we focused our analysis specifically on the group difference at this SOA. First, we found that this unconscious GCE was significantly stronger in the TD group compared to that in the ASD group (Mann–Whitny test, *U* = 118, *P* = 0.010*, effect size = 0.48). Given the extensive evidence supporting the notion that ASD exists on a spectrum, we subsequently divided the TD group into 2 subgroups based on high and low levels of autistic traits. Our objective was to investigate whether the effect of unconscious social attention varies across the spectrum, encompassing different levels of autistic traits ranging from the general population to clinical patients. The interaction of cue congruency and participant group was significant [*F*(2,45) = 3.90, *P* = 0.027*, *η^2^_p_* = 0.15], with the cueing effect only present in the low AQ group [*F*(1,45) = 8.39, *P*_Sidak_ = 0.006*, *η^2^_p_* = 0.16]. As shown in Fig. 2D (left panel), the unconscious GCE of the low AQ group was significantly stronger than that of the ASD group (*P*_Sidak_ = 0.028*), while there was no significant difference between the high AQ and ASD groups (*P*_Sidak_ = 0.135). Similar results were obtained when we selected an equal number of TD participants, matched for gender and age with the ASD participants, and performed the same analysis (see the Supplementary Materials for details). Consistent with previous studies [40,41], these results confirm that autistic people exhibit diminished unconscious gaze-triggered social attention, even when the gaze cues were presented without any salient mask to interrupt their attention. Additionally, although not statistically significant, there was a tendency for the unconscious GCEs to decrease in the high AQ group relative to the low AQ group in general TD adults.

### No evidence of unconscious gaze-triggered social attention in TD children with high autistic traits

The existing literature has consistently emphasized the exploration of how ASD impacts social cognitive development during critical periods, wherein experience exerts a strong influence on brain development [79,80]. To gain deeper insights into the origins of unconscious gaze-triggered social attention, experiment 3 examined its correlation with autistic traits in children who are currently in the critical period of developing social skills through experiential learning [81]. It is worth noting that childhood is also a period with a higher incidence of various neurological disorders [82]. In order to make the task more suitable for children, we replaced the discrimination task with a localization task and utilized only two SOAs of 200 and 600 ms. Additionally, all child participants were divided into two groups based on their levels of autistic traits.

A mixed-design ANOVA (cue congruency × SOA × participant group) found a significant three-way interaction [*F*(1,32) = 5.29, *P* = 0.028*, *η^2^_p_* = 0.14], indicating that the gaze-triggered attentional effect in children varied across participant group and SOA (Fig. 2C). Separate ANOVAs conducted for each SOA condition found that the interaction of cue congruency × group was present at the SOA of 600 ms [*F*(1,32) = 8.72, *P* = 0.006*, *η^2^_p_* = 0.21], but not at 200 ms [*F*(1,32) = 1.59, *P* = 0.216, *η^2^_p_* = 0.05]. Further simple effect analysis on the 600-ms SOA condition revealed a significant attentional effect in the low AQ group [*F*(1,32) = 5.21, *P*_Sidak_ = 0.029*, *η^2^_p_* = 0.14], but not in the high AQ group [*F*(1,32) = 3.58, *P*_Sidak_ = 0.067, *η^2^_p_* = 0.10]. The univariate ANOVA on normalized GCE yielded the same pattern of results (Fig. 2D, right panel). These findings suggest that TD children with low autistic traits possess the unconscious gaze-triggered social attention ability, but this ability is diminished in children with high autistic traits. Furthermore, the unconscious GCE in children with low AQ emerged after 600 ms following the presentation of gaze cues, consistent with the findings in TD adults.

We then merged the data from TD child and adult participants regarding the unconscious gaze-triggered social attention at the SOA of 600 ms to further examine the relationship between such attentional effect and autistic traits. Results revealed a significant negative correlation [*r*(69) = −0.28, *P* = 0.018*]. These findings suggest that TD individuals with higher autistic traits exhibit weaker unconscious gaze-triggered social attention, which aligns with the theory of the continuum distribution of the autism spectrum [83]. Moreover, the correlation coefficients in the children and adults did not differ significantly from each other (Fig. 2E; −0.55 versus −0.27; *z* = −1.39, *P* = 0.165), indicating that the impact of autistic traits on unconscious social attention ability is relatively stable from the childhood to the adulthood.

### Conscious gaze-triggered social attention in children and adults with high autistic traits or ASD

**Fig. 3.**
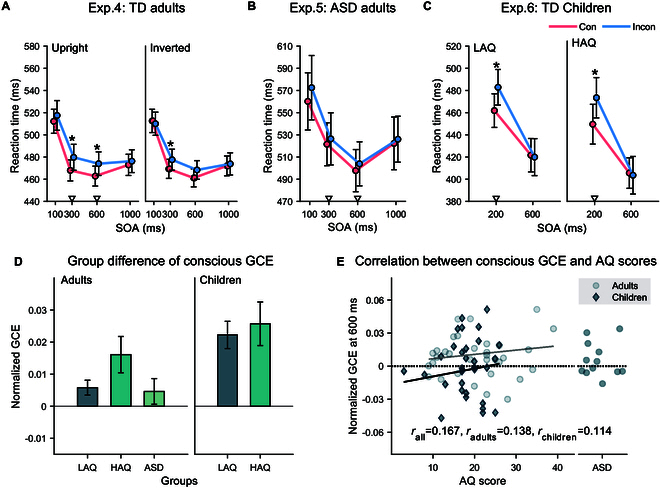
Conscious GCE and its relationship with autistic traits and ASD. (A) When the schematic face was upright (left plane), the visible gaze cues effectively induced a cueing effect and this effect was not significantly affected by face orientation and SOA (right plane). (B) The cue congruency effect was marginally significant in autistic adults. (C) Both the children with low and high autistic traits exhibited a significant cueing effect at the SOA of 200 ms. (D) The bars display the conscious GCEs for adults (collapsed across the SOAs of 300 and 600 ms) and children (at the SOA of 200 ms), showing no significant differences among the groups. (E) The scatterplots demonstrate the correlations between the conscious GCE at the SOA of 600 ms with AQ scores, providing a comparison to the unconscious GCE. The triangle markers in the line graph indicate the levels of SOA used for between-group analysis of the GCEs. Error bars represent SEM. *P < 0.05, corrected for multiple comparisons.

We further examined whether the associations between the unconscious GCE and autistic traits were preserved when the gaze cues were consciously perceived by the participants. As shown in Fig. 3A, the main effect of cue congruency was significant in the TD adults [479 ms versus 485 ms*, F*(1,33) = 8.45, *P* = 0.006**, *η^2^_p_* = 0.20], and no interaction reached significance (all *F*s < 2.3, *P*s > 0.050). These results suggest that the visible gaze cues induced an attentional shift as observed in previous studies [69], and this effect is less susceptible to face inversion and SOA. In the ASD group, the main effect of cue congruency did not reach statistical significance. However, the *P* value suggests a marginal effect, indicating potential evidence of a GCE [Fig. 3B; 525 ms versus 532 ms, *F*(1,11) = 4.57, *P* = 0.056, *η^2^_p_* = 0.29]. Furthermore, there were no significant differences in conscious GCE across the groups (*F*s < 2.0, *P*s > 0.100; Fig. 3D, left panel, illustrates that the GCE collapsed across the SOAs of 300 and 600 ms).

For TD children (Fig. 3C), visible gaze cues triggered a significant attentional orienting effect at the SOA of 200 ms [*F*(1,30) = 33.85, *P* < 0.000***, *η^2^_p_* = 0.53] but not at 600 ms [cue congruency × SOA: *F*(1,30) = 20.63, *P* < 0.000***, *η^2^_p_* = 0.41], and there were no differences on all the GCEs between the low AQ and high AQ groups (*F*s < 2.0, *P*s > 0.100; Fig. 3D, right panel, illustrates the GCE at the SOA of 200 ms). Consistent with previous studies [84], these results suggest that visible gaze cues can trigger attentional orienting. In contrast to observations of the unconscious GCE, the conscious gaze-triggered social attention ability remains relatively intact in both children and adults with high autistic traits or ASD.

Finally, the correlation analysis found no significant correlations between the conscious GCE and AQ scores in TD participants (*P*s > 0.100). The difference between the correlation coefficients in the invisible and visible gaze conditions was significant at the SOA of 600 ms (Fig. 3E; *z* = 2.61, *P* = 0.009**). Taken together, this pattern of results suggests that the unconscious and the conscious gaze-triggered social attention abilities appear to be functionally dissociated, highlighting the critical role of unconscious processing in distinguishing the innate, involuntary component and the acquired, voluntary component of social attention.

## Discussion

Previous studies suggest that social attention contains both the innate, involuntary component and the acquired, voluntary component [47,85], with the former more closely associated with autistic traits [17]. In the current study, we combined the CFF technique with the classical gaze-cueing paradigm to probe the unconscious gaze-triggered social attention. This approach could largely isolate the automatic processing of gaze cues from the influence of consciously acquired strategies. Indeed, the results revealed that both the unconsciously and consciously perceived gaze cues elicited significant attentional effects in TD participants. However, it was only the unconscious gaze-triggered attentional effect that was substantially impaired in children with high autistic traits and adults with ASD and significantly associated with individual autistic traits among TD adults and children. These findings suggest that the unconscious and conscious gaze-triggered attentional orienting can distinguish the innate, involuntary component and the acquired, voluntary component of social attention.

Three key factors have been proposed to contribute to the observed attentional effect triggered by gaze, including innate social modules, postnatal learning through social experience, and physical properties of gaze cues. First, substantial evidence supports the existence of an innate gaze-triggered social attention module in the brain. This module detects the presence of eyes and subsequently orients attention accordingly, without voluntary control [3]. Studies across various animal species have demonstrated their ability to follow gaze directions, a behavior considered a fundamental social attention skill [86], which appears to have emerged early in evolutionary history and remains conserved across species [87]. In humans, research on newborns and infants has revealed their capacity to discriminate [12,88] and respond to eye gaze [89], even when the eyes were presented unconsciously [90]. Furthermore, the impairment of these innate modules has been suggested to contribute to ASD [10]. Specifically, individuals diagnosed with ASD, who inherently exhibit reduced sensitivity to eye gaze [91], may encounter difficulties in extracting the intentions of others from gaze cues. However, providing direct evidence to support this assumption is challenging due to the susceptibility of innate modules to the influence of the other two factors.

Growing evidence supports the idea that social experiences and learning strategies can shape gaze-triggered attention performance. For instance, children raised by blind parents exhibit poorer gaze-following abilities due to their limited exposure to the association between the gaze direction and social intention [27]. Conversely, gaze-triggered attentional orienting can be acquired or improved through extensive training for both TD children [92] and children with ASD [93]. Moreover, the contrast between the iris and the sclera of the averted gaze is particularly salient in human gaze cues. This lower-level physical feature can also elicit attention shifts, even when the facial context is disrupted [94].

Based on comprehensive evidence, we propose a theoretical hypothesis to elucidate the respective contributions of these factors to the unconscious and conscious gaze-triggered social attention found in our study. We speculate that unconscious GCE is mainly supported by innate social modules. First, it operates automatically and unconsciously, but is contingent upon the biosocial context of the schematic face (upright versus inverted), effectively ruling out alternative explanations for its occurrence, such as motor preparation [95,96] or low-level physical shifts [97]. Second, it becomes evident approximately 600 ms after the cue onset. This temporal pattern aligns with the time course of social attention [69,98], suggesting that it may be driven by the distinct social significance conveyed through the eyes [1]. Most importantly, our findings reveal that this effect is impaired in individuals with ASD and shows significantly negative associations with autistic traits in both TD adults and children. Considering the high heritability of ASD [99,100] and autistic traits [101–103], the stable association between this effect and autistic severity at different ages suggests that it is more likely to be supported by the innate abilities. The characteristics of the unconscious gaze-triggered attentional effect imply a high degree of social specificity, meeting most of the criteria for the modularity of cognitive processing [104].

In contrast, the conscious gaze-triggered attentional effect found in the present and previous studies exhibits more complex properties. It occurs earlier (e.g., it is reliably observed at the SOA of 200 ms) and extends over the SOA of 600 ms in adults [69]. Consistent with previous studies, it is not consistently found diminished in adults with ASD [36–38], nor does it demonstrate a clear association with individual autistic traits [105,106]. We speculate that this may be attributed to the influences of other two factors in social attention. On the one hand, the contrast between the iris and the sclera of the averted gaze is more salient in conscious gaze cues than in unconscious gaze cues. On the other hand, according to previous studies, conscious gaze direction can activate broader attention neural network in the ASD group [40], and its degree is positively correlated with AQ scores in TD individuals [107]. These findings indicated that conscious processing of gaze cues could partially relieve and even compensate for the impairment of the innate modules observed in autistic people.

Previous neuroimaging studies have demonstrated that gaze-triggered social attention involves a widely distributed brain network, including the temporal, frontoparietal areas, and brain regions encoding emotional and socio-cognitive information [108]. The dual pathway model posits that the brain network can be divided into two distinct pathways: one fast, ancestral, subcortical pathway, and one slow, developmental, and cortical pathway [109]. Consistent with this model, our results also suggest that the conscious and unconscious parts of this social attention are to some extent dissociable from each other. This dissociation can shed light on the discrepancies in empirical findings and contribute to a comprehensive understanding of the neural underpinnings of ASD.

The literature on consciousness generally agrees that unconscious processes are primarily localized within the local or subcortical neural modules [110]. Based on this consensus, it is reasonable to speculate that the unconscious gaze-triggered attention predominantly relies on the ancestral, subcortical pathway. Indeed, one study has found that the unconscious gaze-triggered attention involves subcortical areas such as the amygdala [111]. However, the activation of these areas appears to be diminished in individuals with ASD [40,112]. These findings suggest that the subcortical module underpinning the unconscious gaze processing may play a crucial role in revealing the social deficits observed in ASD. However, further support from brain imaging research is needed to confirm this inference. It is important to note that the present study only explored the unconscious social attention ability in two age groups, which limits our comprehensive understanding of its developmental trajectory throughout life. Therefore, systematic longitudinal studies are encouraged in the future to provide a complete and precise picture.

In conclusion, the present study reveals a clear distinction between the unconscious and conscious gaze-triggered attentional effects. The unconscious effect, associated with autistic severity, aligns more closely with the functioning of innate social modules. The conscious effect, on the other side, can be influenced by factors such as physical salience and postnatal learning. This differentiation of social attention within the realm of consciousness offers a fresh perspective and approach to discerning between the innate and acquired aspects of social attention. Moreover, we propose that incorporating the unconscious technique with social cognitive paradigms can provide an effective research approach for investigating the social endophenotypes of ASD. This approach has the potential to unveil biomarkers, improve the clinical diagnosis of ASD, and ultimately advance our comprehensive understanding of the socio-cognitive disorders.

## Methods

The research protocols for all experiments were approved by the institutional review board of the Institute of Psychology, Chinese Academy of Sciences. We determined the sample size of each experiment before data collection. All the adult participants and the parents of the child participants provided written informed consent before the experiments and received payment according to the duration of the tasks (50 yuan per hour for adults and 100 yuan per hour for children). The participants (and their parents) were naïve to the purpose of the experiments and were debriefed after the completion of all the experiments and questionnaires. All participants had a normal or corrected-to-normal vision and were not red-green color blind.

We adopted the Chinese version of the Autism Spectrum Quotient (AQ) to estimate the autistic-like traits in TD participants. The AQ contains 50 items and consists of 5 domains, including social skills, communication, imagination, attention to detail, and attention switching. Individuals with higher AQ scores generally possess more autistic traits, which has been demonstrated to be reliable and consistent among Chinese samples at different ages [113,114]. Adult participants and the parents of child participants completed the AQ through an online questionnaire platform. We scored their responses using a binary system, where a mild or strong endorsement of autistic traits is scored as 1 and the opposite response is scored as 0, leading to a maximum score of 50.

### Experiment 1

#### Participants

A priori analysis using G-Power [115] based on a within-subject ANOVA design (*r* = 0.5) suggested that *n* = 36 participants would afford a power of 0.95 for the main effect of cue congruency (congruent versus incongruent) with a medium effect size (*η^2^_p_* = 0.09 [40]). Experiment 1a initially recruited 40 college students. Five of them were excluded, either for not completing the questionnaire or for failing to pass the awareness check task (for detailed information, please refer to the awareness check section). Thus, 35 participants (mean age = 23.03, SD = 2.70, 19 female) with mean AQ scores of 20.31 (SD = 8.86, range = 6 to 41) remained for statistical analysis. For experiment 1b, we recruited another 30 participants with comparable age, gender, and AQ scores (mean age = 23.60, SD = 3.11, 18 females; mean AQ score = 20.30, SD = 6.92, range = 10 to 38). None of them was diagnosed with ASD or other neurodevelopmental disorders.

#### Experimental design

Experiments 1a and 1b adopted a within-subject design, with cue congruency (congruent versus incongruent), face orientation (upright versus inverted), and SOA (100, 300, 600, or 1,000 ms) being the three independent variables. The cue congruency concerned whether the target appeared on the same (congruent) or the opposite (incongruent) side as indicated by the cue.

#### Apparatus and stimuli

Stimuli were presented on a 23.8-inch (1,980 × 1,080 at 60 Hz) LCD monitor using Psychtoolbox extensions [116] for MATLAB (MathWorks Inc., Natick, MA). Participants performed the experimental tasks in a dim-lit room with their heads held in place by a chin rest positioned 57 cm away from the screen. The screen background was yellow (RGB [134, 151, 0]) at a luminance of 34.14 cd/m^2^. A white central cross (0.5° × 0.5°) served as the fixation.

As shown in Fig. 1, the schematic faces (8.6° × 8.6°) were gray (RGB [180, 180, 180]) and consisted of two circular eyes (2° × 2°), a square nose (0.4° × 0.4°), and a straight-line mouth (0.18° × 2.5°). In the upright condition, the geometric center of the face, the nose, and the mouth was placed downward from the screen center by 1°, 1.2°, and 2.8°, respectively. The inverted schematic faces were obtained by mirror-flipping the stimuli vertically with the fixation as the center. In both the upright and inverted conditions, the eyes were positioned horizontally to the left and right sides of the central fixation cross, each at an eccentricity of 2.27°, with the pupils rendered invisible using the CFF technique. Specifically, two anti-phased red-and-green sinusoidal grating discs (1° × 1°, spatial frequency = 4 c/°, mean intensity = 134 for the red channel and = 151 for the green channel) were alternately presented at a frequency of 30 Hz. Because this frequency is beyond the critical chromatic flicker fusion frequency, the two discs were no longer perceived as red-green flickers but as one fused yellow color indistinguishable from the background. The CFF pupil placed corresponding to the morphological structure of averted gaze, namely, apart from the center of the eyes toward left or right by 0.3°. The targets were two white lines (0.8° × 0.15°) tilted either +45° or −45° from the vertical direction.

In experiment 1b, we disrupted the facial morphology by removing the orbit of the eyes and the nose. Two schematic arrow cues, composed of 9 anti-phased red-and-green sinusoidal grating discs (0.3°× 0.3°, spatial frequency = 5 c/°), were positioned in the region of the eyes (see Fig. S1).

#### Procedure

In experiment 1a, each trial began with a central fixation cross. After a random duration (600 to 1,000 ms), a schematic face was presented for a random duration of 500 to 800 ms. The invisible, averted pupils then appeared for 100 ms and disappeared, constituting a gaze toward the left or right direction. The face was presented before the gaze cues to reduce possible attention capture by the central face when it appeared [14]. After a variable interval following the onset of the gaze cue, the target appeared to the left or right side of the face (with an eccentricity of 6°) for 30 ms. The central cross and schematic face remained on the screen until participants responded. Participants were instructed to maintain fixation throughout the entire trial and attend to either side of the face covertly. Upon the target’s appearance, they were instructed to discriminate the orientation of the target (tilted +45° or −45°) and respond as quickly and accurately as possible by pressing the corresponding key (the left key for −45° and the right key for +45°).

The gaze cue was randomly directed toward the left or right with equal likelihood. Meanwhile, the target was presented at either the congruent or incongruent side of the gaze direction, corresponding to 50% validity. To avoid any top-down influence, we instructed the participants that the central face was irrelevant to the task and the target would appear at left or right side with equal likelihood. The upright and inverted face conditions were separated into two sessions with equal trials and presented in a counterbalanced order across participants. In each session, participants completed a minimum of 160 trials, with each combination of cue congruency and SOA randomly and equally distributed across trials. To reduce the experimental duration during the COVID-19 pandemic, 15 participants completed 160 trials, while the other 25 participants completed 192 trials. A short break was provided every 32 trials to avoid fatigue effect.

Before each session, about 30 practice trials were given to familiarize the participants with the task and strengthen their fixation. Additionally, we visually monitored the participants’ eye movements throughout the task and provided reminders if they did not consistently fixate on the cross. During the actual main tasks, all participants fixated on the cross very well and none required reminders. The procedure of experiment 1b closely mirrored that of experiment 1a.

#### Awareness check

Before the experiment, we measured the isoluminance of red and green for the CFF stimuli with the minimal flicker procedure [61]. To further confirm that the flickering of the two chromatic gratings was truly invisible to the participants, both subjective and objective awareness checks were adopted. The participants reported whether they saw anything apart from the schematic face and the target after the experimental task. Additionally, the two-alternative forced-choice task was conducted as an awareness check session. Specifically, the schematic face appeared for a random duration of 500 to 800 ms, and a CFF cue randomly appeared in the left or right eye position of the schematic face with equal chance. The participants were informed that a stimulus would appear in one of the eye positions and that they had to press the left or right key to indicate which eye position contained the stimulus. This task consisted of 2 blocks comprising 40 trials in each of the upright and the inverted conditions and was completed both before and after the main experiment. Participants who reported perceiving CFF stimuli or achieved accuracy levels higher than chance level (50%, binomial test) on the forced-choice task were considered to have failed to pass the awareness test.

### Experiment 2

#### Participants

Experiment 2 focused on group differences in the unconscious gaze-triggered social attention. Therefore, we primarily considered the interaction of cue congruency × participant groups for the sample size estimation. Based on power analysis, we determined that a sample size of *n* = 12 participants in each group would provide a power of 0.95 for a 2 ×3 interaction with a medium effect size (*η*^2^ = 0.11, as reported in [40]). Thirteen adults diagnosed with ASD (10 female, mean age = 22.15 years; SD = 3.72) were recruited through partner hospitals or online forums. All participants provided written diagnostic materials from a qualified clinical facility and had normal IQ scores. We also ruled out other psychiatric disorders through a semistructured interview.

#### Experimental design

Expanding on the findings of experiment 1, experiment 2 examined the unconscious GCE in individuals with ASD, with cue congruency and SOA as the two independent variables. To investigate group differences in the unconscious GCE, we used a mixed-model design with cue congruency as a within-subject factor and participant group as a between-subjects factor. We combined the data from experiments 1 and 2 and divided the participants into three groups (ASD from experiment 2, low AQ and high AQ determined based on the median split of total AQ scores from experiment 1 with the median AQ score = 18.00).

#### Apparatus and stimuli

We used the same apparatus and stimuli as in experiment 1.

#### Procedure

The experimental procedure in experiment 2 was similar to that in experiment 1, except that the ASD group only completed the upright session of the gaze-cueing task and the forced-choice awareness check.

### Experiment 3

#### Participants

We recruited 36 TD children based on the effect size observed in our previous experiments. Two were excluded as they failed to pass the awareness check, leaving 34 participants for further analysis (20 female, mean age = 8.38, SD = 1.83, range = 6 to 12; mean AQ score = 17.79, median AQ score = 17.50, SD = 4.62, range = 3 to 26). They were divided into two groups with high and low autistic traits (median split, *n* = 17 for each group), respectively, and there were no significant differences in age and gender (nonparametric test, *P*s > 0.1) between the two groups. According to the semistructured interview, all the children had not been diagnosed with psychiatric disorders and did not use any medications before the experiment.

#### Experimental design

Experiment 3 used a mixed-model design, with cue congruency and SOA as within-subject factors and participant group as a between-subject factor.

#### Apparatus and stimuli

We used the same apparatus and stimuli as in experiment 1 except that the keyboard was replaced by two large buttons to make the children more comfortable to press. The target stimulus was also replaced by a colored ball (2° × 2°) presented for 300 ms (Fig. 1).

#### Procedure

Children completed a task of striking balls, in which they needed to stare at the central fixation and hit a ball as quickly and accurately as possible when it appeared by pressing the corresponding button (left button if on the left side and right button if on the right side). At the end of each trial, we presented a golden star in the center of the screen as feedback to motivate the children when they pressed the button correctly within 1 s. All children performed 96 trials and took a short break every 16 trials. About 10 practice trials were given to familiarize the children with the task. After the main experiment, they completed a forced-choice awareness check with 40 trials.

### Experiments 4, 5, and 6

#### Participants

These experiments investigated the conscious and voluntary aspects of social attention with sample sizes comparable to the previous three experiments. We recruited 34 TD adults (17 female, mean age = 23.24, SD = 3.24; mean AQ score = 20.82, median AQ score = 21.50, SD = 8.11, range = 9 to 39), 12 ASD adults (8 female, mean age = 24.00, SD = 3.77), and 32 TD children (17 female, mean age = 8.47, SD = 1.76; mean AQ score = 17.94, median AQ score = 18.00, SD = 4.91, range = 3 to 26). Among them, 6 TD adults, 11 autistic adults, and all the TD children participated in the unconscious experiments. They rest for at least 10 min between the two tasks to avoid fatigue effects. There was no significant difference in AQ scores between the TD adults in experiments 1 and 4 [*t*(67) = −0.25, *P* = 0.80, Cohen’s *d* = −0.06].

#### Experimental design

Experiments 4, 5, and 6 had the same experimental design as experiments 1a, 2, and 3, respectively.

#### Apparatus and stimuli

The apparatus and stimuli were almost the same as in experiments 1a, 2, and 3, respectively. The only difference was that the invisible gaze cues were replaced with visible gaze cues (static gray discs with 1° × 1° and RGB [180, 180, 180]) as shown in Fig. 1.

#### Procedure

These experiments had the same procedure as previous experiments, except that there were no awareness check sessions.

### Data analysis

RT was measured from the onset of the target. Only the RT of a correct response within 3 SDs of the mean was considered for further analysis, resulting in the removal of 3.45% of the trials (experiment 1a: 3.24%; experiment 1b: 3.40%; experiment 2: 5.33%; experiment 3: 3.13%; experiment 4: 2.47%; experiment 5: 4.56%; experiment 6: 3.94%). The overall error rate was 1.56% (experiment 1a: 1.63%; experiment 1b: 2.00%; experiment 2: 3.21%; experiment 3: 1.11%; experiment 4: 1.10%; experiment 5: 2.60%; experiment 6: 0.98%), and there was no evidence of a speed–accuracy trade-off. Since there was no significant difference in the error rates between the experimental conditions and participant groups in experiments 1 to 6 (nonparametric test, *P*s > 0.05), we focused on the RTs.

We conducted ANOVA analysis on mean RTs of each participant according to the experimental design using SPSS (version 26). Follow-up simple effect analyses and separate ANOVAs were conducted for significant interaction effects. Furthermore, to clarify the relationship between gaze-triggered attentional orienting and autistic traits, we conducted ANOVA and correlation analysis on the normalized GCE. The normalized GCE was calculated using the difference in the mean RT obtained under the incongruent condition versus that under the congruent condition, then divided by their sum ($\;GCE=\frac{RT_{\text{incong}}-RT_{\text{cong}}}{RT_{\text{incong}}+RT_{\text{cong}}}\;$ ). We used the Fisher r-to-z transformation to compare the difference between the correlation coefficients.

## Data Availability

The data supporting the findings of this study are available at https://osf.io/72egw/. The original experimental code and raw data can be obtained from the corresponding author upon reasonable request.

## Supplementary Materials

Supplementary 1 Supplementary Results Fig. S1
