# Supplementary material for: Unconscious and Conscious Gaze-Triggered Attentional Orienting: Distinguishing Innate and Acquired Components of Social Attention in Children and Adults with Autistic Traits and Autism Spectrum Disorders
Source: Research (Wash D C). 2024 Jul 10;7:0417. doi: 10.34133/research.0417 (PMC11233194; doi:10.34133/research.0417)
Supplement: Supplementary 1 — Supplementary Results Fig. S1 [file research.0417.f1.zip › SupplementaryInformation.docx]

**Supplementary Information**

Supplementary Results:

**No attentional orienting induced by the unconscious arrow cues**

In Experiment 1b, we disrupted the facial morphology of schematic face by removing the orbit of the eyes and the nose. Then, we replaced the pupil discs with schematic arrows comprised of nine CFF discs. Supplementary Figure 1 provides a visual illustration of these modified stimuli. A three-way ANOVA was conducted on the reaction time data, with face orientation, cue congruency, and stimulus onset asynchrony (SOA) as the independent variables. The analysis revealed that only the main effect of SOA was significant [*F* (3,87) = 7.07, *p* < .000^***^, *η^2^_p_* = .20], suggesting the presence of a preparation effect. None of the other main effects or interaction effects reached significance [*F*s < 2.5, *ps* > .100], indicating that the arrow cues presented within the schematic face did not induce significant attentional orienting.

**Controlling for the influence of demographic factors**

To minimize disparities between groups during statistical analysis, we took measures to ensure that an equal number of TD participants were selected and matched for gender and age with the ASD participants, in Experiments 2 and 5. Then we conducted Mix-ANOVA, using cue congruency as a within-subject variable and participant group as a between-subject variable. The analysis revealed a significant interaction between cue congruency and participant group [*F*(2,36) = 4.14, *p* = .024^*^, *η^2^_p_* = .19] for the unconscious condition. Specifically, the unconscious GCE was only observed in the low AQ group [*F*(1,36) = 8.75, *p*_Sidak_ = .005^*^, *η^2^_p_* = .20]. However, no significant group differences were found in terms of GCE under the conscious condition [*F*s < 2.0, *ps* > .100].


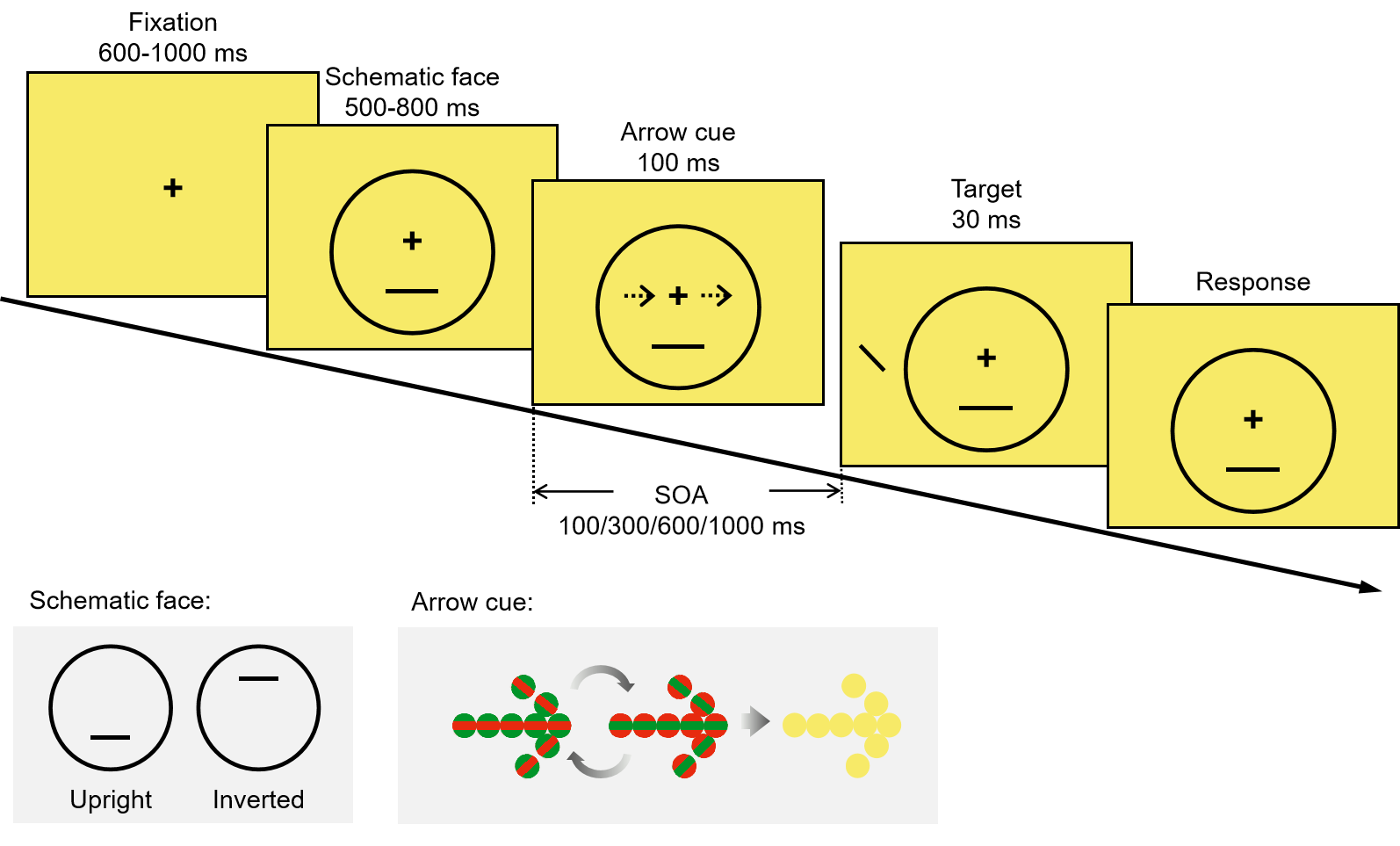


**Supplementary Figure 1**. Illustration of the stimuli and procedures employed in the Experiment 1b. Following the fixation point, a schematic face with intact contour and mouth features was displayed. Then, two arrow cues were presented in the same location as the eye gaze cues in Experiment 1a. Each arrow cue consisted of nine red-and-green sinusoidal grating discs, with five discs arranged horizontally to create the tail of the arrow. Among the remaining four discs, two rotated 45 degrees counterclockwise and two rotated 135 degrees counterclockwise, forming the head of the arrow.
