# Supplementary figures and images for: Unconscious and Conscious Gaze-Triggered Attentional Orienting: Distinguishing Innate and Acquired Components of Social Attention in Children and Adults with Autistic Traits and Autism Spectrum Disorders
Source: Research (Wash D C). 2024 Jul 10;7:0417. doi: 10.34133/research.0417 (PMC11233194; doi:10.34133/research.0417)

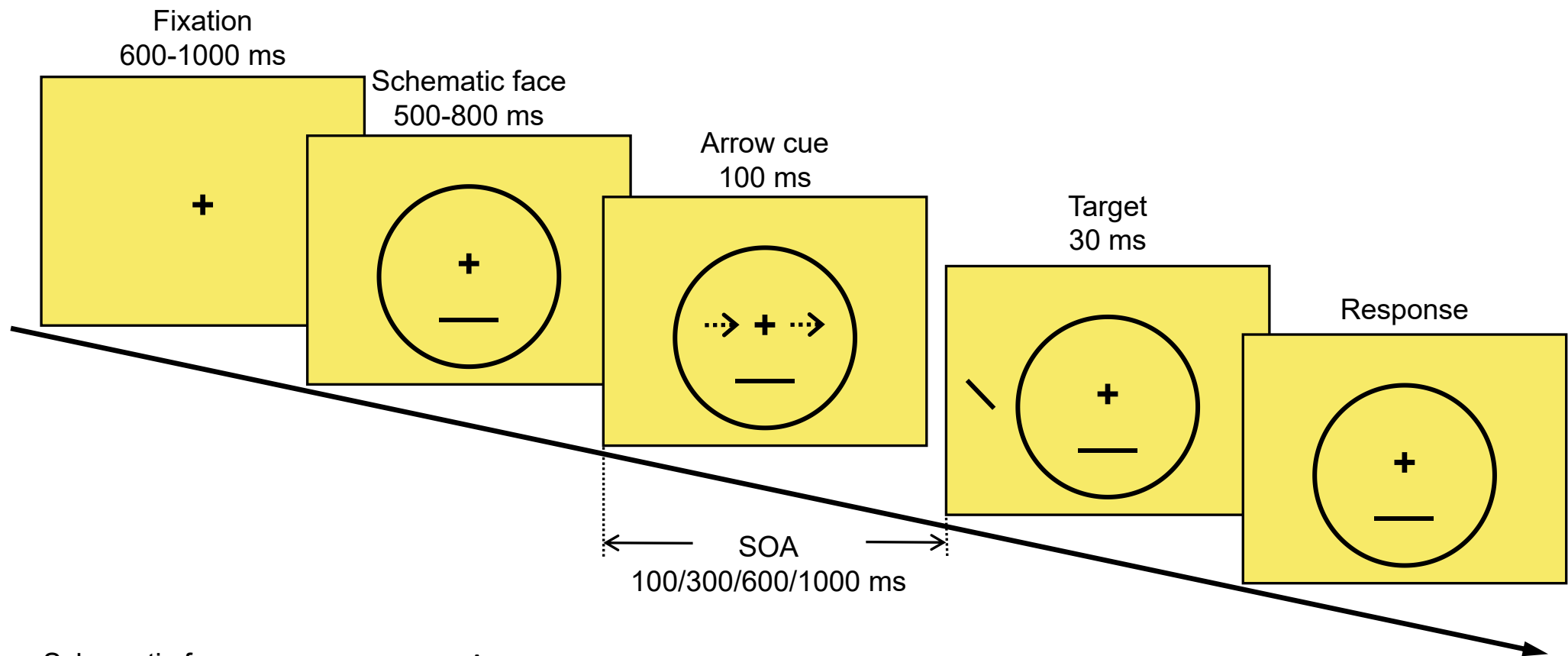

Schematic face:

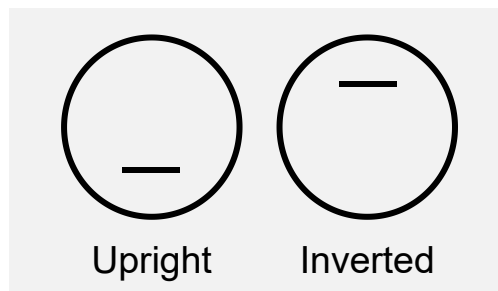

Arrow cue:

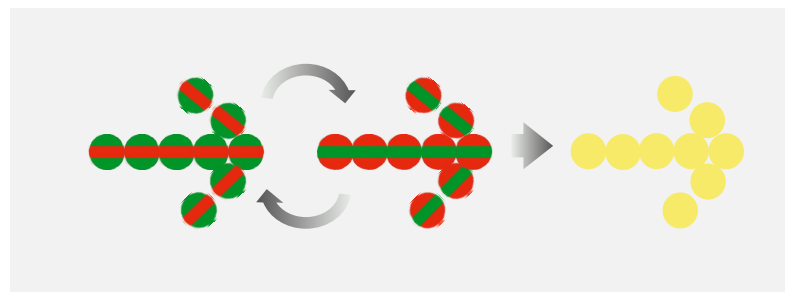

Supplement: Supplementary 1 — Supplementary Results Fig. S1 [file research.0417.f1.zip › SupplementaryFigure1.pdf]
